# Supplementary material for: Physiological Concentrations of Cimicifuga racemosa Extract Do Not Affect Expression of Genes Involved in Estrogen Biosynthesis and Action in Endometrial and Ovarian Cell Lines
Source: Biomolecules. 2022 Apr 5;12(4):545. doi: 10.3390/biom12040545 (PMC9032045; doi:10.3390/biom12040545)
Supplement: Supplementary file 1 [file biomolecules-12-00545-s001.zip › biomolecules-1590063-final-suppl-Table S1-S3.pdf]

Table S1. Concentrations of *Cimicifuga racemosa* (CR) extract used for the gene expression studies.

Cell Line	Concentrations of CR Extract in μg/mL
HEC-1-A	0.005, 0.5, 50
Ishikawa	0.005, 0.5
RL-95-2	0.005, 0.5, 50, 100
KLE	0.005, 0.5, 50, 100
HIEEC	0.005, 0.5, 10, 25
Kuramochi	0.005, 0.5, 50, 100
COV362	0.005, 0.5, 50
OVSAHO	0.005, 0.5, 50
HIO-80	0.005, 0.5, 25, 50

Table S2. Concentrations of *Cimicifuga racemosa* (CR) extract used for Xcelligence experiments in KLE cells.

Replica	Concentrations of CR Extract in μg/mL
1.	400, 300, 200, 100, 50, 20, control
2.	300, 200, 100, 75, 50, 20, control

Table S3. Concentrations of *Cimicifuga racemosa* (CR) extract used for CC_{50} studies.

Cell Line	Concentrations of CR Extract in μg/mL
HEC-1-A	1, 10, 25, 50, 100
Ishikawa	1, 10, 50, 100, 250, 500
RL-95-2	1, 5, 10, 50, 100, 250, 300
KLE	1, 5, 10, 50, 100, 300, 400
HIEEC	1, 5, 10, 20, 30, 50
Kuramochi	1, 10, 50, 100, 150, 250
COV362	1, 10, 25, 50, 75, 100, 200, 300, 400
OVSAHO	1, 10, 50, 100, 250, 500
HIO-80	1, 5, 10, 25, 50, 100, 150
